# Assessment of Health Literacy in Parents of Children With Familial Mediterranean Fever Using the Turkish Health Literacy Scale

**DOI:** 10.7759/cureus.75693

**Published:** 2024-12-14

**Authors:** Ali Öksel, Betül Öksel, Nihal Sahin, Hafize E Sönmez

**Affiliations:** 1 Department of Pediatrics, Kocaeli Medical Park Hospital, Kocaeli, TUR; 2 Department of Pediatric Rheumatology, Kocaeli University, Kocaeli, TUR

**Keywords:** familial mediterranean fever, health literacy, parental education, pediatrics, rheumatology

## Abstract

Background

Health literacy (HL) refers to the ability of individuals to find, understand, and use information and resources to make informed health-related decisions and actions for themselves and others. Managing chronic diseases in children and adolescents requires active family involvement. The primary objective of the study is to evaluate the HL levels of parents of children diagnosed with familial Mediterranean fever (FMF). Secondary objectives include identifying the factors influencing the HL levels.

Materials and methods

Between September 2024 and November 2024, the Turkish Health Literacy Scale-32 (THL-32) was administered to the parents of FMF patients who followed up at the Pediatric Rheumatology Clinic of Kocaeli University Hospital, and their scores were compared based on educational level, residential area, and socioeconomic status.

Results

A total of 81 participants were included in the study, of whom 63 (77.8%) were mothers. Mothers with a university education demonstrated significantly higher HL scores than those with other education levels (p<0.05). However, no significant association was found between fathers' education levels and their THL-32 scores. The total HL score was found to be adequate in 32 parents (39.5%) and excellent in 12 parents (14.8%), indicating that the HL level was sufficient. In the subscale for evaluating information on "treatment and service" and "disease prevention and health promotion," 31 parents (38.3%) had the lowest HL level. A significant correlation was identified between the residential area (urban vs. rural) and THL-32 scores (p=0.034).

Conclusion

Almost half of the parents of our patients had adequate HL. The lowest HL scores were observed in the subscales related to "treatment and service" and "disease prevention and health promotion," particularly regarding the ability to evaluate information. Improving parental HL could lead to better participation in treatment processes and enhanced health outcomes for children with FMF.

## Introduction

Familial Mediterranean fever (FMF) is a hereditary autoinflammatory disorder characterized by recurrent episodes of fever and serositis (peritonitis, pleuritis, acute synovitis), which are self-limiting in nature. The cornerstone of FMF treatment is colchicine. The primary goals of therapy are to reduce the frequency of attacks, improve patients' quality of life, suppress chronic inflammation, and, most importantly, prevent secondary amyloidosis [1,2]. The primary reason for colchicine treatment resistance is non-adherence to therapy. Therefore, it is crucial to educate patients about their disease and emphasize the importance of adhering to the prescribed treatment regimen [3]. Enhancing patients' knowledge about their disease is essential. The World Health Organization defines health literacy (HL) as "the cognitive and social skills that determine the motivation and ability of individuals to access, understand, and use information in ways that promote and maintain good health" [4]. HL is a critical factor in maintaining and promoting health. It serves as a determinant for individuals' health behaviors, health status, and utilization of healthcare services. Inadequate HL is associated with numerous adverse outcomes, including unhealthy lifestyles, lower utilization of preventive healthcare services, higher reliance on curative services, poor treatment adherence, increased hospitalization rates, and higher healthcare costs [5,6]. Limited HL is a significant public health issue worldwide, including in developed countries [6]. Efforts to improve HL have also gained attention in recent years in our country.

Various social and demographic factors, such as education, age, and the social environment of parents, can influence treatment adherence in pediatric FMF patients. One of those factors, parental knowledge, was focused on the studies in pediatric chronic diseases [7-9]. In the literature on pediatric rheumatic diseases, factors such as parental education level, socioeconomic status, and the age of patients directly affect treatment adherence and disease follow-up, making this process crucial from a public health perspective [10].

The HL levels can influence the motivation and ability of parents of children with FMF to access, understand, and utilize information to promote and maintain good health. This study primarily assessed the HL levels in parents of pediatric FMF patients, with the Turkish Health Literacy Scale-32 (THL-32) administered to them. This scale was developed based on the conceptual framework of the European Health Literacy Survey. Secondary objectives involve identifying the factors that influence HL levels, including educational background, socioeconomic status, and residential area.

## Materials and methods

We conducted a cross-sectional study of parents whose children were diagnosed with FMF at the Pediatric Rheumatology Clinic of Kocaeli University Hospital, Kocaeli, Turkey, between September 2024 and November 2024. The inclusion criteria for the study were as follows: being over 18 years of age, being the mother or father of a child aged zero to 18 years with a confirmed diagnosis of FMF for at least six months according to the Tel Hashomer Criteria, having the ability to speak, read, and write in Turkish for the informed consent form and relevant assessment scales, voluntarily agreeing to participate in the study, and providing written informed consent [3]. The questionnaire administered to participants consisted of two sections. The first section gathered data on the FMF-diagnosed child’s demographic information, age, gender, age at diagnosis, disease duration, attack symptoms, medications used, the number of hospitalizations within the past year, and laboratory findings. It also collected information on the parents’ demographic characteristics, including age, education level, occupation, marital status, income level, family history, and place of residence. The second section included questions related to the THL-32 questionnaire (Appendix 1). The questionnaires were completed by the mothers or fathers of the patients.

The Turkish Health Literacy-32 scale was administered to the parents. As a country-specific modification, the dimensions of disease prevention and health promotion in the THL-32 were combined and assessed holistically [11]. This 32-item scale is based on the model developed by the European Health Literacy Survey (HLS-EU) Consortium in 2012 [11]. The THL-32 assesses HL in two main areas: access to, understanding, evaluation, and application of information regarding healthcare and disease prevention/health promotion. Each item is scored on a scale from 1 to 4: very easy, easy, difficult, and very difficult. The scale is generally scored between 0 and 50, and a valid assessment requires at least 80% of the items to be answered. Higher scores indicate a better level of HL. Based on the total score, HL is classified into four levels: 0-25 points represent inadequate, >25-33 points represent problematic, >33-42 points indicate sufficient, and >42-50 points signify excellent health literacy.

This study was approved by the Ethics Committee of Kocaeli University Faculty of Medicine (26.09.2024/397) and conducted in accordance with the Declaration of Helsinki. Informed consent was obtained from the caregivers of all participants.

Statistical analyses were conducted using SPSS Statistics for Windows, Version 15 (Released 2006; SPSS Inc., Chicago, United States). Continuous variables are presented as mean ± standard deviation, while categorical variables are shown as counts (n) and frequencies (percentages). The Kolmogorov-Smirnov test was employed to assess the normality of variable distributions. For group comparisons, the Mann-Whitney U test was applied to compare two groups, and the Kruskal-Wallis test was used for comparisons involving more than two groups. Spearman’s correlation coefficient evaluated associations between variables. A p-value of <0.05 was considered statistically significant.

## Results

During a three-month period, the survey was administered to 81 parents who consented to participate, out of 150 FMF patients attending regular clinic visits. Of the patients, 45 (55.6%) were male. The median age of patients was 10.5 years (2-18), the median age at diagnosis was six years (2-14), and the median disease duration was three years (0.5-16). Regarding attack characteristics, fever was present in 71 (87.7%), abdominal pain in 68 (84%), arthralgia in 37 (45.7%), arthritis in 12 (14.8%), myalgia in 10 (12.3%), chest pain in five (6.2%), erysipelas-like rash in five (6.2%), and orchitis in three (3.7%) patients. The median number of attacks was one (0-8) per year. The median C-reactive protein level was 0.9 mg/L (IQR: 0.3-3.2), and the median erythrocyte sedimentation rate was 5 mm/h (IQR: 4-10.5). Urinary proteinuria was absent in 76 (93.8%) patients, while five (6.2%) patients had nephritic-level proteinuria. Table 1 details demographic and clinical characteristics of the patients.

**Table 1:** Demographic and clinical characteristics of the patients

Characteristics	Median (25p-75p) or n(%)
Female	36 (44.4%)
Male	45 (55.6%)
Age of onset of complaints (years)	5 (3-8)
Age of diagnosis (years)	6 (4-14)
Delay in diagnosis (years)	1 (1-2)
Disease duration (years)	3 (0.5-6)
Number of attacks per year	1 (0-2)
Hospitalization in the last year	0 (0-0)
Fever	71 (87.7%)
Abdominal pain	68 (84%)
Chest pain	5 (6.2%)
Arthritis	12 (14.8%)
Arthralgia	37 (45.7%)
Eryzepel-like rash	5 (6.2%)
Orchitis	3 (3.7%)
Myalgia	10 (12.3%)
C-Reactive protein	0.9 (0.1-31)
Erythrocyte sedimentation rate	5 (1-30)
Normal urine analysis	76 (93.8%)
Nephritic proteinuria	5 (6.2%)
Presence of accompanying disease	12 (14.8%)
Surgery history	0
Allergy	5 (6.2%)
Treatment	
Colchicine	69 (85.2%)
Colchicine opocalcium	6 (7.4%)
Anakinra	2 (2.5%)
Kanakinumab	4 (4.9%)

All patients received colchicine, with six (7.4%) receiving opocalcium as the colchicine preparation. Four patients (4.9%) received canakinumab, and two (2.5%) received anakinra. Of the parents who completed the questionnaires, 63 (77.8%) were mothers, and 18 (22.2%) were fathers. The average age of mothers was 38.4±5.5 years, and of fathers, it was 42.1±5.9 years. While 60% of fathers had completed secondary or high school, most mothers (65.5%) had either completed elementary/middle school or high school. Only five mothers (6.2%) had incomplete primary education. Among mothers, 57 (70.4%) were housewives. Among fathers, 47 (58%) were workers, and 18 (22.2%) were self-employed. Regarding socioeconomic status, 41 (50.6%) cases lived in a district. A total of 75 (92.6%) patients had health insurance. Regarding economic status, 48 (59.3%) of parents described their financial situation as balanced (income equals expenditure). In family medical history, 12 (14.8%) patients had a parental consanguinity, and 46 (56.8%) had a family history of rheumatic diseases. Table 2 details the parents' education, occupational status, familial socioeconomic status, and medical history.

**Table 2:** Overview of parents' educational backgrounds, occupational statuses, as well as the family's socioeconomic characteristics and relevant medical history

Variables	
Average maternal age (years)	38.4±5.5
Average paternal age (years)	42.1±5.9
Parents filling out the survey	
Mother	63 (77.8%)
Father	18 (22.2%)
Maternal education level	
Incomplete primary education	5 (6.2%)
Primary school	19 (23.5%)
Middle school	14 (17.3%)
High school	20 (24.7%)
University	23 (28.4%)
Paternal education level	
Incomplete primary education	0 (0%)
Primary school	13 (16%)
Middle school	19 (23.5%)
High school	30 (37%)
University	19 (23.5%)
Mother's profession	
Housewife	57 (70.4%)
Worker	8 (9.9%)
Civil Servant	8 (9.9%)
Teacher	8 (9.9%)
Father's profession	
Worker	47 (58%)
Freelance	18 (22.2%)
Retired	5 (6.2%)
Civil servant	4 (4.9%)
Teacher	3 (3.7%)
Farmer	3 (3.7%)
Doctor	1 (1.2%)
The area of residence	
District	41 (50.6%)
Metropolitan area	28 (34.6%)
Provincial center	9 (11.1%)
Village	3 (3.7%)
Economic status	
Balanced (Income equals expenditure)	48 (59.3%)
Income less than their expenses	18 (22.2%)
Income exceeding their expenses	15 (18.5%)
Parental consanguinity	12 (14.8%)
Presence of rheumatological disease in the family	
Familial Mediterranean fever	46 (56.8%)
Rheumatoid arthritis	37 (45.7%)
Acute rheumatic fever	5 (6.2%)
Behçet's disease	1 (1.2%)
Juvenile idiopathic arthritis	1 (1.2%)
Systemic lupus erythematosus	1 (1.2%)

The overall THL-32 score for parents was 33.8±6.2, indicating adequate HL. The general HL levels were classified as inadequate in nine (11.1%) parents, problematic/limited in 28 (34.6%), adequate in 32 (39.5%), and excellent in 12 (14.8%). There was no difference in THL-32 scores between mothers (33.74±7) and fathers (34±6.75) who filled out the questionnaire (p=0.69).

In the subscale for healthcare services, the lowest HL was observed in the appraising information, with 31 parents (38.3%) demonstrating deficiencies. The highest performance, however, was applying and understanding knowledge, with 22 parents (27.2%) scoring excellent. Similarly, in the disease prevention and health promotion subscale, the weakest HL was observed in the appraising information, with 31 parents (38.3%) demonstrating deficiencies. Conversely, the best HL was in understanding information, with 17 parents (21%) demonstrating excellence. HL levels for THL-32 subscales are given in Table 3.

**Table 3:** The levels of health literacy of parents of children with children with familial Mediterranean fever on the THL-32 subscales THL-32: Turkish Health Literacy Scale-32

Subscales	Inadequate	Problematic-limited	Adequate	Excellent
Total score	9 (11.1%)	28 (34.6%)	32 (39.5%)	12 (14.8%)
In the field of treatment and service				
Accessing information	10 (12.3%)	29 (35.8%)	24 (29.6%)	18 (22.2%)
Understanding information	10 (12.3%)	27 (33.3%)	22 (27.2%)	22 (27.2%)
Appraising information	31 (38.3%)	29 (35.8%)	15 (18.5%)	6 (7.4%)
Applying information	11 (13.6%)	22 (27.2%)	26 (32.1%)	22 (27.2%)
Disease prevention and health promotion				
Accessing information	14 (17.3%)	33 (40.7%)	24 (29.6%)	10 (12.3%)
Understanding information	16 (19.8%)	35 (43.2%)	13 (16%)	17 (21%)
Appraising information	31 (38.3%)	33 (40.7%)	9 (11.1%)	8 (9.9%)
Applying information	20 (24.7%)	34 (42%)	18 (22.2%)	9 (11.1%)

The total THL-32 score was compared to fathers' and mothers' education status (into which parents filled out the questionnaire). University-educated mothers had significantly higher scores than other groups (p=0.02; university vs. elementary school p<0.001; university vs. middle school p<0.001; university vs. high school p=0.01). No significant difference was found in the total scores based on fathers' education levels. The THL-32 score was a significant difference based on the residential area. The average THL-32 score was 25.4±10.9 in villages compared to 36.4±7 in metropolitan areas (p=0.034). The total THL-32 score did not differ with the families' economic and health insurance status (Table 4). No correlation was found between parental THL-32 total scores and disease duration, diagnostic delay, annual attack frequency, hospitalization in the past year, C-reactive protein levels, or erythrocyte sedimentation rate (Table 5).

**Table 4:** Comparison of the THL-32 total score according to the change in the parents' socioeconomic status THL-32: Turkish Health Literacy Scale-32

THL-32 total score			
Variables	N (%)	Mean ± SD	p
Mother’s education level*			
Incomplete primary education	5 (7.9%)	31.4 ±8.2	0.02 (vs. university)
Primary school	12 (19%)	29.9±3.8	<0.001(vs. university)
Middle school	12 (19%)	30±6.9	<0.001(vs. university)
High school	19 (30%)	34.3±5.5	0.01(vs. university)
University	15 (24.1%)	40.7±4.7	-
Father’s education level*			
Primary school	4 (22.2%)	27.2±7.1	>0.05 (vs. others)
Middle school	1 (5.5%)	36.5	>0.05 (vs. others)
High school	5 (27.7%)	35±3.7	>0.05 (vs. others)
University	8 (44.6%)	36.5±6.8	>0.05 (vs. others)
Place of residence			
Village	3 (3.7%)	25.4±10.9	0.034 (vs. metropolitan area)
District	41 (50.6%)	32.4±6.1	>0.05 (vs. others)
Provincial center	9 (11.1%)	34.7±5.9	>0.05 (vs. others)
Metropolitan area	28 (34.6%)	36.4±7	0.034 (vs. village)
Economic status			
Income less than their expenses	18 (22.2%)	31.1±6.2	>0.05 (vs. others)
Balanced (income equals expenditure)	48 (59.2%)	34.3±6.7	>0.05 (vs. others)
Income exceeding their expenses	15 (18.6%)	35.3±7.8	>0.05 (vs. others)
* A comparison was made between the parents who completed the survey and whether they were mothers or fathers.			

**Table 5:** Correlation analysis between the THL-32 total scores and characteristics of the disease THL-32: Turkish Health Literacy Scale-32

Correlated variables		Spearman’s rho	p
Parental THL-32 total scores	Disease duration	-0.08	0.50
	Diagnostic delay	-0.15	0.19
	Annual attack frequency	-0.07	0.56
	Hospitalization in the past year	-0.1	0.39
	C-reactive protein levels	0.04	0.72
	Erythrocyte sedimentation rate	0.01	0.92

## Discussion

The findings of this study indicate that parents of pediatric FMF patients exhibit adequate health literacy (HL) levels, with a median total score of 33.33 (IQR: 28.65-37.76) based on the THL-32 questionnaire. These results are significant given the critical role of parental HL in managing chronic diseases like FMF, as adequate HL is associated with improved understanding of disease management, adherence to treatment protocols, and better clinical outcomes in children. Consistent with the literature, higher HL levels in caregivers have been positively correlated with enhanced health behaviors and outcomes, underscoring the importance of interventions aimed at improving HL to optimize treatment efficacy and overall well-being in pediatric patients. According to data released by the Ministry of Health of the Republic of Turkey, the HL level of seven out of every 10 individuals in Turkey is low [12]. Although only one of the ten parents had an excellent HL level, more than half of the parents had adequate or excellent HL levels in our study. These HL levels are reasonable compared to the reporting of the Ministry of Health of the Republic of Turkey. The region we are located in has a relatively higher socio-economic level compared to the rest of the country, which may explain the higher HL scores observed in our study compared to the general population of Turkey.

Our results showed that the subscale appraising health information was the most inadequate in treatment, service, disease prevention, and health promotion. Similarly, in studies conducted in different countries, appraising health information was the most inadequate subscale in adults with chronic diseases and the general adult population [13,14]. Researchers note that overwhelming online data can confuse appraising health information. To address this, they suggest implementing strategies to filter out irrelevant information and recommend that healthcare providers effectively guide individuals in finding and interpreting relevant information [15].

The World Health Organization defines non-medical factors, such as education and income, which influence health outcomes, as the social determinants of health [16]. In population-wide studies, inadequate HL was linked to low socioeconomic status and income and education disparities [6,14]. In 269 families of Turkish routine healthy children, the parental role and HL were assessed using the THL-32 scale. HL was found to increase significantly with higher levels of education [17]. This finding is consistent with studies in the literature that show raising parents' knowledge, attitudes, and perceptions leads to a decrease in permanent disabilities and child mortality rates due to unintentional injuries in children and adolescents. These findings suggest that targeted educational campaigns, especially aimed at parents with lower education levels, could effectively improve health literacy and, consequently, enhance child health outcomes [18]. Our results showed that income, health insurance, and the father's education status did not influence HL. However, mothers who graduated from university exhibited higher levels of HL than others. Additionally, when the data was based on the place of residence, we observed that poor parental HL was predominantly present among families living in villages, in contrast to those residing in metropolitan areas.

A previous study assessing the disease knowledge of families with FMF patients showed that mothers were more involved with their children and knew more about FMF than fathers. The study found a positive correlation between the parents' knowledge level and educational status. The findings may be due to the fact that the current study included a very limited number of fathers [19]. However, in another study, educational status did not affect parental HL in juvenile idiopathic arthritis (JIA) [20]. In our results, most survey respondents (77.8%) were mothers, indicating that the parents accompanying the outpatient clinic visit were primarily mothers. Half of the mothers in our study were university or high school graduates, and most were housewives. In contrast, 60% of the fathers held university or high school diplomas, and nearly all were employed. Unlike the mothers, the HL scores of the fathers did not vary based on their education levels. These findings suggest that information exchange may occur within the social networks of working men, regardless of their educational backgrounds. Encouraging mothers more actively involved in caring for pediatric FMF patients to participate in social and professional networks may help improve their health literacy levels.

Untreated or poorly treated FMF can result in increased morbidity and mortality, mainly due to amyloidosis. A study evaluated medication adherence among 82 FMF patients using the MASIF scale and found no evidence that the socioeconomic status of families affected medication adherence [21]. Our study did not examine medication adherence, which is a limitation of our research. Instead, the THL-32 score did not correlate with data indirectly indicating disease course, such as the annual attack frequency, hospitalizations in the past year, C-reactive protein levels, or erythrocyte sedimentation rate in our study. However, we plan to conduct a prospective study to investigate the effects of parental HL on the management of FMF.

The main limitations of our study were the relatively small sample size, data collection from a single center, and the inability to directly analyze the clinical findings of the children.

## Conclusions

The findings underscore significant inadequacies in health literacy, particularly in the subscales of "treatment and service" and "disease prevention and health promotion," especially regarding the ability to evaluate information. These results emphasize the importance of targeted strategies to improve parental health literacy, with a focus on parents with lower educational levels and parents from rural backgrounds. Enhancing health literacy in this population could foster more effective participation in treatment processes and contribute to better health outcomes for children with FMF.

## Appendices

Appendix 1 

**Table 6:** Turkey Health Literacy Scale-32 (THL-32)

No	How easy/difficult would it be to do the following, from very easy to very difficult?	Very easy	Easy	Difficult	Very difficult
1.	When you have a health complaint, investigate and find out if it is a symptom of a disease				
2.	When you have a health complaint, read and understand any text (brochure, booklet, poster, etc.) on this subject				
3.	When you have a health complaint, consider whether the advice of your family or friends is reliable				
4.	When you want to go to a health institution, you need to research and find out which doctor you should see				
5.	Research and find out how to apply (such as making an appointment) when you want to go to a health institution				
6.	When you want to go to a health institution, make an appointment by phone or internet				
7.	Research and find information about treatments for diseases that concern you				
8.	Understanding your doctor's explanations about your illness				
9.	Evaluate the advantages and disadvantages of different treatment options suggested by your doctor				
10.	Use your medications as recommended by healthcare professionals (such as doctors and pharmacists)				
11.	Understand the instructions on the medicine box for using the medicine				
12.	Deciding whether you need a second opinion from a different doctor				
13.	Understanding information about pre-test/examination preparations (such as dieting)				
14.	Searching for and finding the location of the unit you want to reach in the hospital (laboratory, polyclinic, etc.)				
15.	Deciding what to do in an emergency (such as an accident or sudden health problem)				
16.	Calling an ambulance when necessary				
17.	Have your health monitored and checked at regular intervals as recommended by your doctor				
18.	Researching and finding information about conditions that may be harmful to your health, such as being overweight or having high blood pressure				
19.	Understanding health warnings about conditions that may be harmful to your health, such as being overweight or having high blood pressure				
20.	Seeking out information on how to deal with unhealthy behaviors such as smoking and insufficient physical activity				
21.	Understanding health warnings about how to deal with unhealthy behaviors such as smoking and insufficient physical activity				
22.	Research and find information about health screenings you should have based on your age, gender and health status (such as screenings for breast diseases for women and prostate-related diseases for men)				
23.	Understanding the recommended actions to be taken to be healthier from sources such as the internet, newspapers, television, and radio				
24.	Deciding whether the information suggested for being healthier in sources such as the internet, newspapers, television, and radio is reliable or not				
25.	Understanding information on food packaging that may affect your health				
26.	Evaluating the positive and negative features of the environment you live in (such as your home, street, neighborhood) that affect your health				
27.	Finding information about what can be done to make your living environment (such as your home, street, neighborhood) healthier				
28.	Evaluate which of your daily behaviors (such as exercising, eating healthy, not smoking) affect your health				
29.	Changing your lifestyle (such as exercising, eating healthy, not smoking) for your health				
30.	Being able to apply the diet list given in writing by the dietitian				
31.	Advising your family or friends on how to be healthier				
32.	Interpreting health policy changes				
